# Association between blood‐based protein biomarkers and brain MRI in the Alzheimer’s disease continuum: A systematic review

**DOI:** 10.1002/alz.095097

**Published:** 2025-01-09

**Authors:** Micaela Mitolo, Gemma Lombardi, Riccardo Manca, Benedetta Nacmias, Annalena Venneri

**Affiliations:** ^1^ IRCCS Istituto delle Scienze Neurologiche di Bologna, Bologna, Italy Italy; ^2^ University of Parma, Parma Italy; ^3^ University of Florence, Florence, Italy Italy; ^4^ Brunel University London, Uxbridge United Kingdom; ^5^ Department of Neuroscience, Psychology, Drug Research and Child Health, University of Florence, Florence, Florence Italy; ^6^ Brunel University London, London United Kingdom

## Abstract

**Background:**

Blood‐based biomarkers are becoming emerging tools, easily detectable and minimally invasive, to reveal neurodegeneration and neuroinflammation in Alzheimer’s disease (AD). However, a comprehensive and up‐to‐date overview of the association between blood‐based biomarkers and brain parameters as measured by MRI is not available. The aim of this review is to fill this gap and clarify the relationship between the main peripheral blood‐based protein biomarkers (i.e., Aβ, p‐tau, t‐tau, NfL and GFAP) and brain parameters derived from MRI in the AD continuum.

**Method:**

A literature search was carried out searching the PubMed and Web of Science databases for articles published up to May 2023. Articles were excluded according to the following criteria: no data on protein biomarkers; no data on the association between biomarkers and MRI data; articles not presenting novel data; articles that included only cognitively unimpaired older adults or patients with other neurological conditions; animal studies; molecular imaging studies; non‐peer reviewed articles; non‐English language articles; case reports.

**Result:**

A total of 33 articles were included in this systematic review. The findings revealed the following: hippocampal volume was positively correlated with Aβ42 and Aβ42/Aβ40 and negatively with Aβ40 plasma levels; p‐tau181 and p‐tau217 concentrations were negatively correlated with temporal grey matter volume and cortical thickness; NfL levels were negatively correlated with white matter microstructural integrity, whereas GFAP levels were positively correlated with myo‐inositol values in the posterior cingulate cortex/precuneus. Moreover, higher plasma GFAP levels were also negatively associated with hippocampal atrophy, lower cortical thickness, and smaller white matter volumes in temporal and parietal regions.

**Conclusion:**

Our systematic review showed strong associations between the main blood‐based protein biomarkers and MRI markers of AD, highlighting a high degree of concordance between these measurements. This suggests a possible advantage in combining multiple AD‐related markers to improve accuracy of early diagnosis, prognosis, progression monitoring and treatment response.

**Acknowledgement**: This study is supported by funding obtained under the National Recovery and Resilience Plan (NRRP), Mission 4 Component 2 Investment 1.3 ‐ Project code PE0000006, CUP D93C22000930002, “A multiscale integrated approach to the study of the nervous system in health and disease” (MNESYS).

